# Unproven stem cell therapy for macular degeneration

**DOI:** 10.18632/oncotarget.21891

**Published:** 2017-10-19

**Authors:** Zubair A. Ansari, Ajay E. Kuriyan, Thomas A. Albini

**Affiliations:** Thomas A. Albini: Department of Ophthalmology, University of Miami, The Bascom Palmer Eye Institute, Miami, FL, USA

**Keywords:** stem cell, age-reated macular degeneration, retinal detachment, food and drug administration, intravitreal injection

Currently, there are multiple attempts to utilize stem cells to potentially reverse or stabilize vision loss resulting from retinal degenerative disorders, the most common of which is age-related macular degeneration (AMD). Schwartz et al. conducted the first small, multicenter phase 1/2 open-label human case series utilizing human embryonic stem cells in patients with end stage, non-exudative AMD [1]. Though longitudinal results of this study are pending, early results from this trial demonstrated that the cells derived from a modified embryonic stem cell line could be transplanted without the feared complication of tumor development and showed some promising signs of clinical efficacy.

The success of studies such as this have increased public awareness, enthusiasm, and hope for stem cell therapies, purported to treat and/or cure dozens of diseases. A multibillion dollar industry already exists charging patients directly for stem cell therapies, often offered by practitioners without appropriate experience treating the disease of concern, and circumventing much of traditional, mainstream medicine and US Food and Drug Administration (FDA) regulations. Consequently each year tens of thousands of patients are charged for unproven stem cell therapies without documented efficacy or safety. These clinics have been financially successful, particularly in the treatment of joint disorders and are expanding into other therapeutic areas including ocular diseases.

Last March, we reported a case series of three patients who underwent bilateral, same day, intravitreal injection of autologous adipose tissue-derived “stem cells” for AMD not regulated by the FDA [2]. The consequences of this for-cash procedure were devastating, with nearly all eyes in the three patients developing epiretinal membranes and delayed severe retinal detachments, resulting in visual acuities ranging from 20/200 to no light perception in the better eye at one year of follow-up - which included multiple surgeries for retinal detachment. Visual acuities in the better seeing eye prior to the procedures had ranged from 20/30 to 20/50.

A similar case of intravitreal “stem cell” injections at a different, unaffiliated stem cell clinic with blinding complications, including severe retinal detachments has been recently reported [3]. The patient received the treatment for exudative macular degeneration and experienced vision loss from 20/200 in the right eye and 20/400 in the left, prior to the procedure, to hand motion in the right eye and light perception in the left, after the procedure [3]. A third report documented poor outcomes including retinal detachment after subretinal injection of stem cells at a third facility [4]. Together, these cases establish that the scope of the problem with unproven stem cell therapy for retinal disease is not limited to one site.

The “stem cell” clinics employ direct-to-consumer advertisements using websites that emphasize patient testimonials, which can result in patients not being properly informed of the potential complications and the paucity of rigorous peer-reviewed publications demonstrating efficacy data for these procedures. A 2016 study found 187 unique “stem cell” clinic websites offering interventions at 215 clinics [5].

“Stem cell” clinics that use autologous stem cells, such as the ones described above, claim they are minimally manipulating cells applied for homologous use, and that consequently by law these therapies should not fall under strict FDA regulatory oversight. FDA draft guidance statements narrowing the definition of minimal manipulation [6] and clarifying homologous use [7] were created in order to eliminate any doubt that the use of autologous “stem cells” should fall under the regulatory oversight of the FDA.

The FDA has recently posted a warning letter to the facility that treated the three patients described above. In the accompanying press release [8], FDA Commissioner Scott Gottlieb, M.D. explains that “stem cell clinics that mislead vulnerable patients into believing they are being given safe, effective treatments that are in full compliance with the law are dangerously exploiting consumers and putting their health at risk.” He goes on to say that the FDA will be increasing enforcement actions against these clinics offering unproven stem cell therapies. Seemingly the FDA’s interpretation of the pertinent laws will have to be tested in the courts. If the FDA’s interpretations hold up, they will help protect patients from “stem cell” clinics that provide untested, potentially dangerous treatments. Enforcement can serve to help draw a clear distinction between the unregulated “stem cell” clinics and ethical and bona fide scientific clinical research in cellular therapies, with the ultimate goal of FDA approved, proven stem cell treatments.
